# Copper Materials for Caries Management: A Scoping Review

**DOI:** 10.3390/jfb15010010

**Published:** 2023-12-23

**Authors:** Veena Wenqing Xu, Mohammed Zahedul Islam Nizami, Iris Xiaoxue Yin, John Yun Niu, Ollie Yiru Yu, Chun-Hung Chu

**Affiliations:** 1 Faculty of Dentistry, University of Hong Kong, Hong Kong, China; u3008489@connect.hku.hk (V.W.X.); mnizami@forsyth.org (M.Z.I.N.); irisxyin@hku.hk (I.X.Y.); niuyun@hku.hk (J.Y.N.); ollieyu@hku.hk (O.Y.Y.); 2 Department of Mineralized Tissue Biology and Bioengineering, The Forsyth Institute, Harvard University, Cambridge, MA 02138, USA

**Keywords:** copper, materials, caries, prevention, antibacterial, antimicrobial

## Abstract

This study comprehensively reviewed the types, properties and potential applications of copper materials for caries management. Two researchers independently searched English publications using PubMed, Scopus and Web of Science. They screened the titles and abstracts of publications presenting original studies for review. They included 34 publications on copper materials, which were categorized as copper and copper alloy materials (13/34, 38%), copper salt materials (13/34, 38%) and copper oxide materials (8/34, 24%). All reported copper materials inhibited the growth of cariogenic bacteria such as *Streptococcus mutans* and *Candida albicans*. The materials could be doped into topical agents, restorative fillers, dental adhesives, drinking water, dental implants, orthodontic appliances, mouthwash and sugar. Most publications (29/34, 83%) were laboratory studies, five (5/34, 14%) were animal studies and only one paper (1/34, 3%) was clinical research. In conclusion, copper and copper alloy materials, copper salt materials and copper oxide materials have an antimicrobial property that inhibits cariogenic bacteria and *Candida albicans*. These copper materials may be incorporated into dental materials and even drinking water and sugar for caries prevention. Most publications are laboratory studies. Further clinical studies are essential to validate the effectiveness of copper materials in caries prevention.

## 1. Introduction

Dental caries is a widespread, globally prevalent chronic oral disease. According to the World Health Organization (WHO) report, nearly half of the population suffers from untreated caries worldwide [1]. Dental caries is the fourth most expensive illness to treat, causing a serious worldwide disease burden. Individuals may suffer from caries at all stages of their lives. The etiologic factors associated with caries are cariogenic microbes, host or tooth surface, substrate and time [2]. The colonized cariogenic microbes on tooth surfaces can metabolize fermentable carbohydrates and generate organic acids. Enamel and dentin can be dissolved in acids, even though they are highly mineralized hard tissues [3]. Continuous mineral loss causes destruction of the tooth structure and ultimately results in dental caries [4]. To achieve caries management, the keys lie in suppressing the activity of bacteria and the growth of biofilms to inhibit acid production and further reduce demineralization. Therefore, researchers have developed various dental materials with antimicrobial properties to prevent caries.

Among metallic dental materials, silver nanoparticles and silver compounds are widely used. Because silver ions have strong antimicrobial properties, various silver-containing compounds are synthesized and used in dentistry, such as silver diamine fluoride [5]. Although silver materials exhibit outstanding bactericidal effects, the staining effect of silver ions affects the appearance and dissatisfies patients [6]. Additionally, silver materials can display inherent cytotoxicity in oral cells even at a low concentration [7]. These disadvantages of silver materials limit their clinical application. Therefore, researchers want to develop novel dental materials with good anticaries properties and no staining effect.

Replacing silver with copper is a potential solution to avoid staining. As a common metal element, copper has a long history of being used widely in different areas. Copper has been used to treat and prevent illnesses since ancient times, according to the Smith Papyrus, dated between 2600 and 2200 B.C. [8]. Practices such as the administration of copper preparations to address skin diseases, syphilis and tuberculosis have also been documented over the past few centuries [9]. These documents highlighted the ability of copper to kill bacteria with low toxicity to human cells.

**Figure 1:**
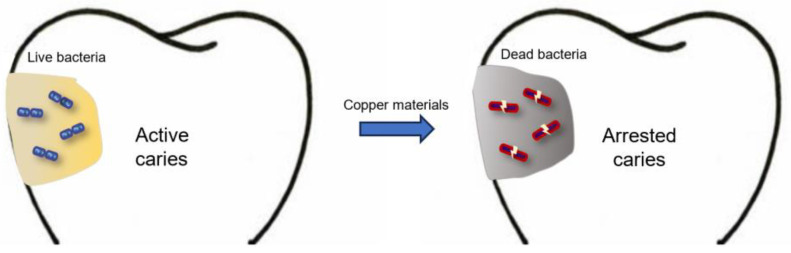
Effects of copper materials on carious lesions.

In anticaries applications, copper has received a great deal of interest in recent decades. Back in the 1980s, scientists doped copper materials into topical agents and drinking water to investigate the anticaries effect of copper [10,11]. In recent years, various copper-based nanomaterials with antibacterial properties for caries treatment and prevention have emerged with the development of nanotechnology [12]. Copper-based nanoparticles show excellent antibacterial capabilities due to the high surface-area-to-volume ratio of the nanostructure [13]. Researchers have incorporated copper nanoparticles into dental restorative materials, implants and oral health products for the prevention of oral diseases. In addition, several copper compounds have been developed for caries management, such as copper salt, copper oxide and copper alloy. These copper-based materials have been developed for their anticaries properties and have shown auspicious results. Figure 1 illustrates the effects of copper materials on carious lesions. However, our search indicated no reviews on the use of copper materials for caries management. This work aims to review and summarize the copper materials used for controlling dental caries in a comprehensive way. This outline may aid in creating novel copper materials for caries management in future studies.

## 2. Methods

### 2.1. Search Strategy

Two independent investigators performed a literature search in three common databases (PubMed, Scopus and Web of Science) to identify publications. The keywords were (copper OR Cu) AND (caries OR tooth decay OR demineralisation OR demineralization OR remineralisation OR remineralising OR remineralization OR remineralizing). The search was limited to publications in English, with no restrictions on the publication date. The last search was conducted on 1 September 2023. 

### 2.2. Study Selection and Data Extraction

**Figure 2:**
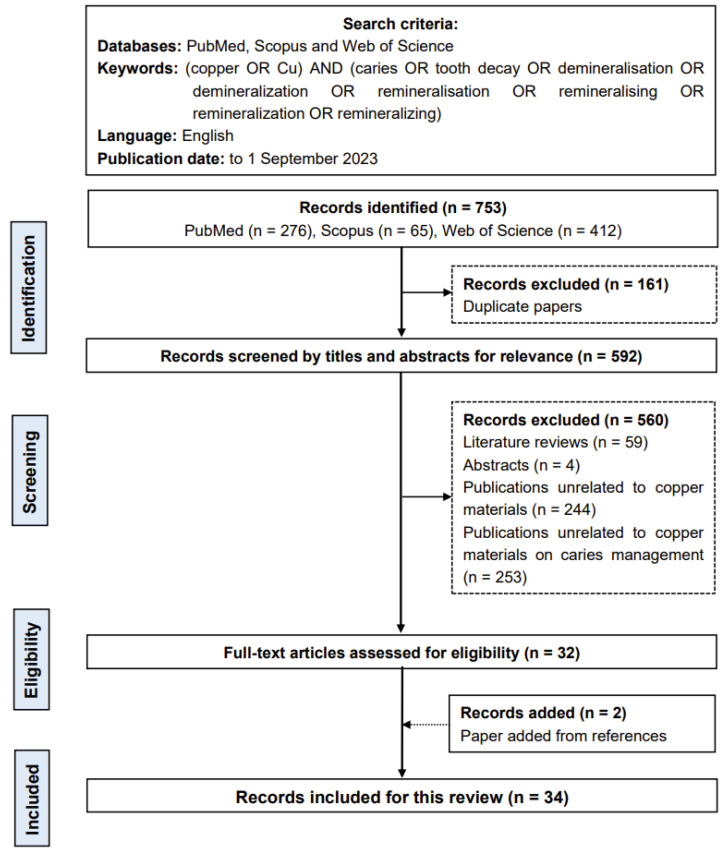
Flow chart of the literature search.

This scoping review includes original studies focusing on the copper materials used for caries management (Figure 2).

Two researchers independently examined and eliminated duplicate publications from the three databases to create a list of publications. They screened the titles and abstracts of the publications to identify potentially qualifying publications. They excluded literature reviews, studies unrelated to copper materials, studies unrelated to copper materials on caries management and other irrelevant studies. The two researchers then retrieved the entire texts of the remaining publications for review. They chose publications in which the developed copper materials were the fundamental components for caries management. After that, they manually screened the reference lists of the selected publications to choose eligible publications. They discussed this with another researcher to determine the publications included in this review. They recorded the information of the publications, including the authors, year, journal and issues, the copper materials used, the study design, the anticaries properties studied and the potential applications of the copper materials.

### 2.3. Assessment of Risk of Bias

Two investigators independently assessed the risk of bias in individual studies. The evaluation was based on previous systematic reviews [4,14]. They assessed the quality of each study using seven parameters, which were: (1) presence of a control, (2) amount of material for assessment, (3) exposure time, (4) material characterization, (5) biocompatibility assessment, (6) blinding of observers and (7) sample size justification. A low risk of bias was assigned to publications that reported six or seven items. Publications reporting three to five items were regarded as medium risk, while those with fewer than three items were considered high risk.

## 3. Results

The preliminary literature search found 753 potentially eligible publications (276 publications in PubMed, 65 publications in Scopus and 412 publications in Web of Science). The two researchers removed 161 duplicate records. After screening the titles and abstracts, 560 publications were excluded because they were literature reviews, publications unrelated to copper materials, publications unrelated to copper materials on caries management or other irrelevant studies. A search of the references of the selected publications yielded two publications that matched the inclusion criteria. As a result, this review comprised 34 publications.

**Figure 3:**
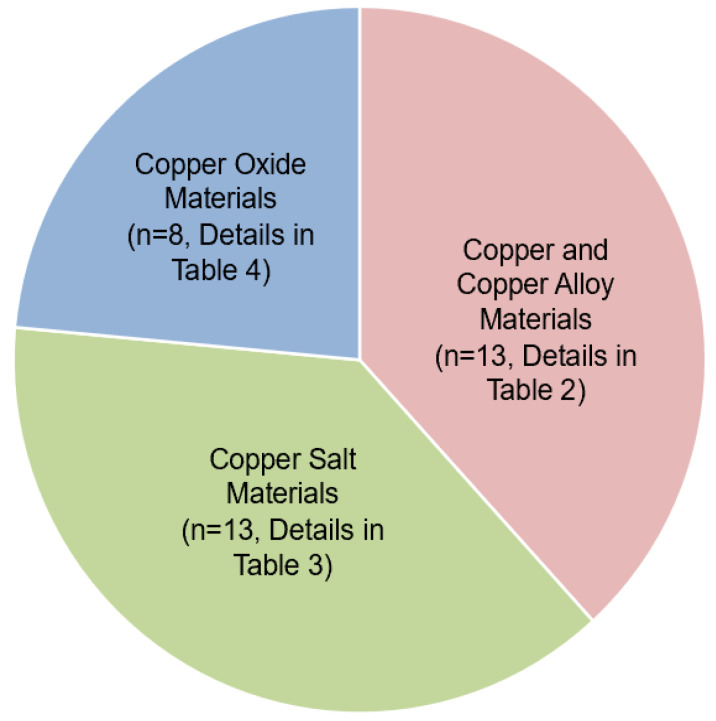
Number of publications of the three groups of copper materials for caries management.

The risk of bias in the 34 publications was examined (Table 1). A total of 8 publications had a low risk of bias, whereas 26 publications had a medium risk of bias. Of the 34 publications included, the majority (29/34, 83%) were in vitro studies, 5 (5/34, 14%) were animal studies and only 1 publication (1/75, 3%) was a clinical study. The copper materials could be doped into topical agents (8/34, 24%), restorative fillers (6/34, 18%), dental adhesives (6/34, 17%), drinking water (2/34, 6%), dental implants (1/34, 3%), orthodontic appliances (1/34, 3%), orthodontic brackets (1/34, 3%), mouthwash (1/34, 3%) and sugar (1/34, 3%). Other publications (8/34, 23%) did not mention specific uses. The copper materials involved in these publications were classified as copper and copper alloy materials (13/34, 38%), copper salt materials (13/34, 38%) and copper oxide materials (8/34, 24%). The three categories of copper materials developed for caries management are depicted in Figure 3.

## 4. Discussion

**Figure 4:**
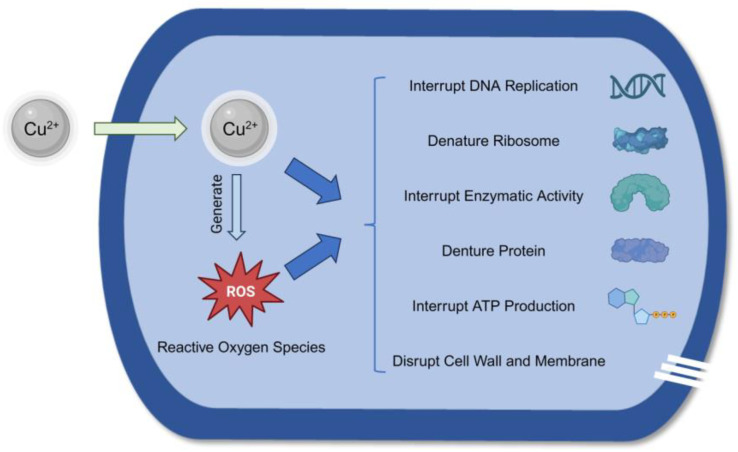
The antimicrobial mechanism of copper ions.

All the copper materials included in this review exhibited antimicrobial properties. Copper materials can breach the microorganisms’ cell walls and enter the cell. Then, the released copper ions can hinder the metabolic functions of the bacteria, such as denaturing proteins and interrupting enzymatic activity and deoxyribonucleic acid replication, and can even result in cell death [47,48]. Figure 4 summarizes the antimicrobial mechanism of copper ions. 

### 4.1. Copper and Copper Alloy Materials

Copper exists as elemental copper in copper and copper alloy materials. Elemental copper is a pure copper material with no valence change, but it has significant antibacterial properties. Table 2 summarizes 13 publications on copper and copper alloy materials for caries management. One publication is an animal study, and the other publications are in vitro studies. 

#### 4.1.1. Copper Nanoparticles

Nanotechnology is a contemporary research trend that has been particularly beneficial in developing diverse nanomaterials utilized in various applications [49,50]. Nanoparticles consist of nanometer-sized materials (1 to 100 nm). They have exceptional mechanochemical and biological features, such as a high surface-to-volume ratio, high strength and stability, high solubility and chemical reactivity and promising antibacterial capabilities. As a result, different nanoparticles, including copper nanoparticles, have been produced for caries control with promising outcomes [49].

Copper nanoparticles exhibit antimicrobial effects on various microbes. They have been incorporated with various other materials, and they have been developed for different potential applications. Copper nanoparticles can be used alone as the active component in caries control. 

Copper nanoparticles are used alone in caries management. One study incorporated copper nanoparticles with commercial glass ionomer cement [17]. The authors reported that the composites demonstrated an antibacterial effect against *Streptococcus mutans* (*S. mutans*) and *Streptococcus sanguinis* (*S. sanguinis*) and did not have cell cytotoxicity. 

Copper nanoparticles are more commonly used when incorporated with metal active substances in caries management. One group doped copper nanoparticles into bioglass and combined the bioglass with silver diamine fluoride as a topical agent [21]. Combining the copper bioglass with silver diamine fluoride increased the viscosity up to three times while lowering the flowability. Additionally, the copper bioglass in silver diamine fluoride exhibited lower cytotoxicity and a better antibacterial effect against *Staphylococcus aureus* (*S. aureus*) and *S. mutans*. Another study combined copper nanoparticles with silver nanoparticles and metronidazole in glass ionomer cement [34]. The result suggested that the composite materials effectively inhibited the growth of *S. mutans* and *S. aureus* compared to conventional glass ionomer cement. 

**Table 2:** Studies of copper and copper alloy materials against cariogenic bacteria *.

Copper (Cu) Material [Ref.]	Bacteria	Design	Intervention (Optimal and Range of Concentration Used) and Control(s)	Potential Use
Cu nanoparticles [20]	*S. mutans*	In vitro	Intervention: 10 μg/mL graphene oxide-Cu nanocomposites Controls: graphene oxide; Cu nanoparticles; water	-
Cu nanoparticles [31]	*S. mutans*	In vitro	Intervention: 60 μg/mL Cu-chitosan nanoparticles Controls: chlorhexidine; Cu nanoparticles; water	Topical agent
Cu nanoparticles [42]	*S. mutans*	In vitro	Intervention: 5% ZnO + (0.2%, 0.1% or 0.2%) Cu nanoparticles Control: water	Dental adhesive
Cu nanoparticles [33]	*S. mutans*	In vitro	Intervention: 1.25% and 2.5% Cu nanocomposites with ZnO and F Control: water	Dental adhesive
Cu nanoparticles [18]	*S. mutans*	In vitro	Intervention: (0.25%, 0.05–0.25%) Augmentin-Cu nanoparticles Control: water	Restorative filler
Cu nanoparticles [45]	*S. mutans*	Animal	Intervention: 256 μg/mL ZnO-Cu-doped Rose Bengal nanoparticles Controls: chlorhexidine; water	Topical agent
Cu nanoparticles [21]	*S. mutans* *, S. aureus*	In vitro	Intervention: (10%, 1–10%) Ag(NH_3_)_2_F-Cu nanoparticle-doped bioglass Control: water	Topical agent
Cu nanoparticles [34]	*S. mutans, S. aureus*	In vitro	Intervention: 0.5% Thymus vulgaris-Cu nanoparticles Controls: Ag nanoparticles; metronidazole; water	Restorative filler
Cu nanoparticles [17]	*S. mutans, S. sanguinis*	In vitro	Intervention: (4%, 1–4%) Cu nanoparticles Control: water	Restorative filler
Cu-Ni nanoparticles [26]	*S. mutans, S. aureus, E. coli*	In vitro	Intervention: (1000 μg/mL, 0.01–1000 μg/mL) Cu-Ni nanoparticles Controls: nanoparticles; Ni nanoparticles; water	-
Cu-Ti alloy [35]	*S. mutans*	In vitro	Intervention: Cu-Ti alloy Controls: Ti alloy; water	-
Cu-Ti alloy [16]	*S. mutans, P. gingivalis*	In vitro	Intervention: Cu-Ti alloy Controls: Ti alloy; water	Dental implant
Copper-Iron alloy [43]	*S. mutans, S. sanguinis*	In vitro	Intervention: (4.5 wt.%, 2.5–4.5 wt.%) Cu-bearing stainless steel Control: stainless steel	Orthodontic appliances

* The systematic search yielded no clinical studies on copper oxide materials for caries management.

Various publications investigated the incorporation of zinc and copper nanoparticles. One study created a unique nanocomposite composed of copper-doped zinc peroxide nanoparticles and the antibacterial chemical Rose Bengal [45]. They demonstrated that the nanocomposite could kill *S. mutans* swiftly and efficiently using a chemodynamic treatment. The nanocomposite could also reduce the pathogenicity of *S. mutans* by reducing acid production and inhibiting the synthesis of extracellular polysaccharides. 

Another study doped zinc oxide and copper nanoparticles into dental adhesive and applied the adhesive to the dentin surface [42]. The researchers reported that adding zinc oxide and copper nanoparticles could increase the antimicrobial activity and ultimate tensile strength without affecting the resin–dentin bond strength or nanoleakage. Some researchers prepared fluoride-containing zinc oxide and copper oxide nanocomposites. The results indicated that, compared with conventional adhesives, the adhesive with the nanocomposites exhibited an antibacterial effect against *S. mutans* while retaining the mechanical qualities of the bonded restorations. 

Some other studies combined copper nanoparticles with nonmetal materials. Researchers fabricated graphene oxide-copper nanocomposites, maintaining a long-term release of copper nanoparticles to obtain a continuous antimicrobial effect [20]. The researchers discovered that the biomass of *S. mutans* biofilm was considerably reduced when the nanocomposites were used. They also found that the nanocomposites could disrupt biofilm architecture, impede exopolysaccharide synthesis and transport and dysregulate the expression of exopolysaccharide-associated genes. 

One study synthesized hybrid nanoparticles comprising copper nanoparticles with a chitosan shell. The researchers found that the antibacterial effectiveness of the hybrid nanoparticles against *S. mutans* was comparable to that of oral antimicrobial drugs such as chlorhexidine and cetylpyridinium chloride. Furthermore, chitosan may interact with both the tooth hydroxyapatite and the bacterial cell wall, improving copper adherence to the tooth surface and amplifying its antibiofilm effect. 

Another study prepared antibiotic Augmentin-coated copper nanoparticles [18]. The results demonstrated that the anticaries ability of the experimental composite was higher than that of the control group without cell cytotoxicity. Furthermore, the release kinetics of the composite exhibited a steady and slow release of copper nanoparticles, showing a sustained antibacterial effect.

#### 4.1.2. Copper Alloy Materials

A copper alloy is a metallic solid made up of copper and other metals or nonmetals. Using different elements can result in different alloy phases, which can modify the alloy’s properties to fulfill different purposes [51]. Alloys may exhibit unique magnetic, optical, physicochemical and antibacterial properties compared with pure monometallic materials [52]. In recent years, many studies have investigated different bimetallic copper alloys for dental caries management.

One study prepared bimetallic Cu-Ni nanoparticles via a simple chemical method, and it reported that the bimetallic copper alloy exhibits an antibacterial effect against *S. mutans*, *S. aureus* and *Escherichia coli* (*E. coli*) [51]. Some researchers created the gradient Cu-Ti alloy and investigated the antibacterial effect of the alloy and the mechanism by which the alloy inhibits bacterial growth [35]. The authors demonstrated that the Cu-Ti alloy exhibited significant bactericidal activity against sessile bacteria and efficient biofilm-restrained ability by down-regulating biofilm-related genes. 

Another group also prepared a Cu-Ti alloy for caries control and developed it into a dental implant [16]. The study demonstrated that the alloy showed antibacterial effectiveness against *S. mutans* and *Porphyromonas gingivalis* (*P. gingivalis*) with acceptable biocompatibility. Some other investigators doped copper into stainless steel to prepare a Cu-Fe alloy for orthodontic appliances [43]. The investigators reported that the alloy could offer a fresh approach to preventing caries during orthodontic treatment by efficiently inhibiting the adhesion, development, activity and metabolism of *S. mutans* and *S. sanguinis* biofilms.

### 4.2. Copper Salt Materials

Copper salt refers to a compound in which all cations are copper ions and are combined with anions through ionic bonds [53]. Copper salts usually exist as solutions and primarily rely on copper ions for their chemical characteristics. Copper salts are widely utilized and can be used for sterilizing, pest control and other purposes. Copper sulfate (CuSO_4_), copper fluoride (CuF_2_), copper chloride (CuCl_2_) and copper iodide (CuI) are frequently employed to manage dental caries. Table 3 summarizes 13 publications on copper salt materials for managing dental caries. Four of the publications were rat studies, one was a clinical trial and the other publications were in vitro studies on copper materials for caries management.

#### 4.2.1. Copper Sulfate Materials

Copper sulfate has long been employed as an antibacterial drug, and it has been used as an anticaries agent since 1984 [54]. Some researchers collected plaque from incipient occlusal fissures and then treated the plaque topically with copper sulfate [10]. The results demonstrated that the copper sulfate solution showed a significant difference compared to the control group in the survival rate of *S. mutans*. 

One paper used varied concentrations of a copper sulfate solution as a topical agent on dental enamel [37]. The investigators reported that the copper sulfate solution at a concentration of 5 mmol/L could stop *S. mutans* from growing and could stop acids from breaking down the enamel. Another study also looked into the effect of a copper sulfate solution on the prevention of enamel demineralization [25]. The researchers set up a double-blind randomized clinical trial and found that copper sulfate together with fluoride exhibited a strong ability to protect the enamel from demineralization. 

Furthermore, a group published two similar rat studies on the antibacterial effect of copper sulfate [11,23]. A copper sulfate solution was administered via drinking water in these two studies and exhibited the ability to inhibit *S. mutans* growth. Another study employed a rat model to assess the effect of incorporating copper sulfate in sucrose by co-crystallization [24]. The investigators fed the rats sucrose with various levels of copper sulfate and examined the caries scores of the rats. The findings showed that rats fed sucrose along with copper sulfate had lower levels of *Streptococcus sobrinus* (*S. sobrinus*). The researchers also proposed that co-crystallizing copper sulfate into sucrose may be an effective strategy to lessen the cariogenic potential of sucrose. 

Copper phosphate can also exhibit an antibacterial effect and be incorporated into dental materials. An article investigated the ion release from copper phosphate restorative filler and the effect of copper phosphate on *S. mutans* growth [38]. The researchers reported that copper ions could be released from the copper phosphate cement and demonstrated a great antibacterial effect. 

#### 4.2.2. Copper Halide Materials

Copper halides, such as copper fluoride, copper chloride and copper iodide, are also used as anticaries agents. In one study, copper fluoride and copper sulfate were administered topically to hamsters to examine the anticaries properties of these two substances [36]. As a result, copper fluoride had a larger antibacterial effect than copper sulfate. 

One study developed a novel copper chloride iontophoresis device and applied copper chloride to intact teeth [28]. The researchers measured copper chloride’s anticaries effectiveness using the DIAGNOdent pen, and the results showed that copper chloride performed substantially better than the control group did. 

Three publications investigated copper iodide nanoparticles developed for caries management. In these three studies, the researchers incorporated polyacrylic acid-coated copper iodide nanoparticles into dental adhesives and restorative fillers [15,30,44]. They proved that after adding the copper iodide nanoparticles, the dental adhesives exhibited an antibacterial effect against *S. mutans* and *Lactobacillus acidophilus* (*L. acidophilus*) while maintaining effective shear bond strength and low cytotoxicity. 

#### 4.2.3. Other Copper Salt Materials

A study developed copper complexes of the hydrochloride salt of 3-chlorobenzaldehyde hydralazine hydrazone (C_30_H_20_N_8_Cl_2_Cu) and tested the antimicrobial effect of the copper complexes with *S. mutans*, *Enterococcus faecalis* (*E. faecalis*) and *Candida albicans* (*C. albicans*) [27]. The result showed that the copper complexes had excellent inhibitory activity in these microbes. 

### 4.3. Copper Oxide Materials

Researchers also created copper oxide materials for caries management. Table 4 summarizes eight publications on copper oxide materials for managing dental caries. All were in vitro studies, and the systematic search yielded no clinical or animal studies on copper oxide materials for caries management. 

#### 4.3.1. Copper Oxide Materials

Ordinary copper oxide has already been doped into various dental materials. One study evaluated several dental adhesives with the incorporation of different components. The outcome demonstrated that copper-containing adhesives significantly reduced the biofilm formation of *S. mutans* [41]. Another study evaluated the anticaries properties of a copper oxide-containing restorative filler on the root surface [39]. The researchers suggested that the restorative filler exhibited antibacterial activity against *S. mutans*.

With the development of nanotechnology, researchers started to synthesize copper oxide nanoparticles from copper oxide for caries management. Some researchers examined the inhibiting effect of copper oxide nanoparticles against various oral microbes [40]. They found that copper oxide nanoparticles could strongly inhibit bacteria such as *S. mutans*, *L. acidophilus* and *Lacticaseibacillus casei* (*L. casei*), as well as fungi such as *C. albicans*, *Candida krusei* (*C. krusei*) and *Candida glabrata* (*C. glabrata*). 

The literature search revealed that combining copper oxide nanoparticles with other materials was used in more investigations than just employing copper oxide nanoparticles alone. Some researchers applied coatings of copper oxide nanoparticles and zinc oxide nanoparticles onto orthodontic brackets to decrease the risk of caries during the orthodontal treatment [29]. The results suggested that the composites significantly reduced the growth of *S. mutans* around the orthodontic brackets. One article reported the novel zinc oxide-copper oxide nanocomposites and evaluated their anticaries abilities [46]. The authors assessed the characterization of the nanocomposites, and they proved that the material had an antibacterial effect against *S. mutans*. In another study, the researchers combined hydroxyapatite, copper oxide and titanium dioxide and developed nanocomposites [19]. They examined the antibacterial ability of the nanocomposites and found that the material could inhibit the growth of *S. mutans*. In addition, copper oxide nanoparticles can be combined with chitosan and doped within dental adhesive to exhibit an antibacterial effect against *S. mutans* and *Lactobacilli acidophilus* (*L. acidophillus*) [32]. 

**Table 4:** Studies of copper oxide materials against cariogenic microorganisms *.

Copper Material [Ref.]	Microorganism	Intervention (Optimal and Range of Concentration Used) and Control(s)	Potential Use
CuO cement [41]	*S. mutans*	Intervention: commercial CuO cement Control: conventional cement without CuO	Dental adhesive
CuO cement [39]	*S. mutans*	Intervention: commercial CuO cement Control: conventional cement without CuO	Restorative filler
CuO nanoparticles [46]	*S. mutans*	Intervention: 47.5% Cu, CuO nanoparticles with or without F Control: water	-
CuO nanoparticles [19]	*S. mutans*	Intervention: 40 mg/mL hydroxyapatite, 1.5 mg/mL CuO, 3.2 mg/mL TiO_2_ Control: water	-
CuO nanoparticles [29]	*S. mutans*	Intervention: CuO nanoparticle-coated brackets Controls: ZnO-coated brackets; brackets without CuO nanoparticles	Orthodontic brackets
CuO nanoparticles [32]	*S. mutans, L. acidophillus*	Intervention: 50 μg/mL CuO nanoparticles with or without chitosan Controls: chitosan; water	Dental adhesive
CuO nanoparticles [40]	*S. mutans, L. acidophilus, L. casei, C. albicans, C. krusei, C. glabrata*	Intervention: (1000 μg/mL, 1–1000 μg/mL) CuO nanoparticles Control: water	-
Cu_2_O nanoparticles [22]	*S. mutans, S. aureus, E. coli*	Intervention: 1% Bi_12_O_17_Cl_2_ + 2% Cu_2_O nanoparticles Control: water	Topical agent

* The systematic search yielded no clinical or animal studies on copper oxide materials for caries management.

#### 4.3.2. Cuprous Oxide Materials

Cuprous oxide has reducing properties and can be oxidized to copper oxide. A research group developed a hydrogel membrane doped with bismuth oxychloride and cubic cuprous oxide nanoparticles. In their study, they tried to achieve tooth-whitening and bacterial-inhibition effects through photodynamic therapy [22]. The researchers proved that the novel hydrogel membrane had tooth-whitening and antibacterial abilities against *S. mutans* under green light. 

Although we have various copper materials used for caries management, most publications are laboratory studies. These materials can be categorized as copper and copper alloy materials, copper salt materials and copper oxide materials. They inhibit the growth of cariogenic bacteria and *Candida albicans*. Furthermore, the antibacterial properties of copper materials are sometimes not as good as those of other materials, and the substrate and application are limited. Although biocompatibility is one of the most important points in the studies on biomaterials, almost two-thirds of the studies did not report the biocompatibility (cytotoxicity) of copper or copper composite materials.

This review found that only a few studies have good quality with a low risk of bias. Therefore, more quality studies are necessary to report the use of copper materials in caries research. In addition, in vivo studies are essential to validate the effectiveness of copper materials in caries management. Moreover, copper materials may be developed with stronger antibacterial properties and doped with more kinds of materials for a greater variety of applications in caries management.

## 5. Conclusions

The most common types of copper materials for caries management are copper and copper alloy materials. According to the literature, copper materials have antibacterial properties. Copper materials can be incorporated into restorative fillers, topical agents, dental adhesives, mouthwash and even drinking water and sugar for caries prevention. Most publications are laboratory studies, and more in vivo studies are essential to validate the effectiveness of copper materials in caries management. 

## Figures and Tables

**Table 1:** Risk of bias in the 34 publications included.

First Author, Year [Ref.]	Items of Assessment							Score	Risk of Bias
	Control Group	Material Quantity	Exposure Time	Sample Size	Material Characterization	Biocompatibility	Observer Blinding		
Sabatini, 2015 [15]	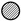	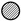	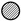	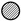	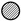	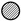		6	Low (6–7)
Liu, 2016 [16]	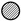	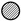	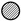	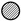	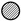	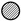		6	Low (6–7)
Aguilar-Perez, 2020 [17]	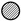	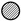	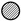	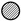	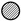	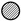		6	Low (6–7)
Pasha, 2020 [18]	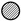	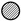	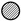	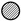	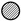	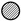		6	Low (6–7)
Imani, 2021 [19]	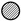	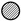	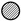	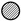	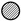	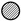		6	Low (6–7)
Mao, 2021 [20]	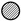	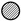	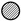	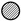	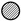	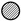		6	Low (6–7)
Bang, 2022 [21]	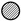	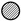	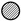	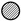	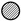	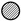		6	Low (6–7)
Li, 2022 [22]	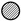	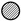	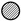	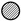	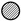	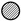		6	Low (6–7)
Afseth, 1984 [23]	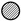	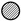	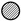	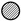		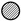		5	Medium (3–5)
Afseth, 1984 [11]	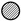	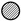	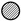	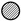		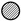		5	Medium (3–5)
Rosalen, 1996 [24]	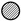	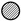	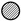	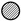		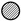		5	Medium (3–5)
Abdullah, 2006 [25]	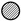	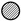	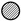	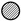			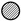	5	Medium (3–5)
Argueta-Figueroa, 2014 [26]	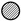	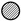	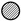	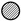	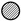			5	Medium (3–5)
Bakale, 2014 [27]	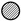	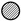	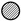	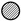	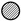			5	Medium (3–5)
Girenes, 2014 [28]	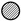	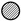	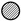	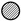	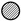			5	Medium (3–5)
Ramazanzadeh, 2015 [29]	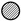	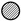	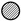	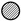	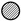			5	Medium (3–5)
Renné, 2017 [30]	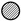	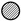	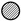	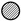	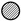			5	Medium (3–5)
Covarrubias, 2018 [31]	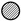	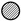	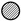	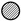	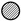			5	Medium (3–5)
Javed, 2021 [32]	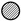	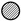	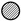	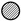	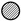			5	Medium (3–5)
Altankhishig, 2022 [33]	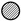	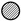	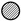	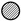	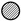			5	Medium (3–5)
Ashour, 2022 [34]	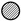	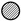	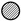	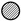	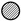			5	Medium (3–5)
Fan, 2022 [35]	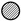	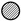	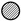	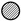	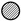			5	Medium (3–5)
Meiers, 1984 [10]	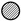	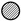	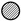	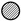				4	Medium (3–5)
Maltz, 1988 [36]	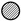	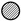	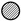			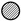		4	Medium (3–5)
Brookes, 2003 [37]	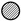	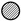	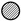	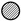				4	Medium (3–5)
Foley, 2003 [38]	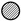	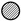	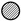	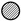				4	Medium (3–5)
Thneibat, 2008 [39]	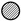	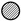	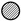	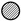				4	Medium (3–5)
Amiri, 2017 [40]	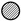	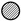	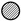	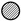				4	Medium (3–5)
Glauser, 2017 [41]	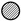	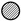	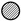	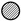				4	Medium (3–5)
Gutiérrez, 2019 [42]	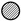	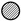	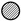	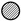				4	Medium (3–5)
Lan, 2022 [43]	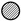	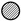	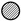			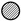		4	Medium (3–5)
Mennito, 2022 [44]	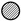	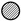	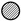	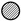				4	Medium (3–5)
Zhang, 2022 [45]	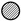	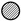	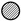		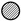			4	Medium (3–5)
Matsuda, 2019 [46]	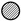	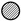	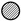					3	Medium (3–5)

**Table 3:** Studies of copper salt materials against cariogenic microorganisms.

Copper Material [Ref.]	Microorganism	Design	Intervention (Optimal and Range of Concentration Used) and Control(s)	Potential Use
C_30_H_20_N_8_Cl_2_Cu solution [27]	*S. mutans, E. faecalis, C. albicans*	In vitro	Intervention: (50 μg/mL, 0.19–50 μg/mL) C_30_H_20_N_8_Cl_2_Cu solution Controls: C_30_H_21_N_8_Cl_3_Zn; C_31_H_24_N_8_OCl_2_Ni; water	-
CuSO_4_ solution [10]	*S. mutans*	In vitro	Intervention: (1.0 mM, 0.5–1.0 mM) CuSO_4_ solution Control: water	Topical agent
CuSO_4_ solution [37]	*S. mutans*	In vitro	Intervention: (10 mM, 5.0–10 mM) CuSO_4_ solution Control: water	Topical agent
CuSO_4_ solution [25]	*S. mutans*	Clinical	Intervention: 1.25 mM CuSO_4_ solution Controls: amine fluoride; water	Mouthwash
CuSO_4_ solution [23]	*S. mutans*	Animal	Intervention: (5.0 mM, 0.1–5.0 mM) CuSO_4_ solution Controls: NaF solution; water	Drinking water
CuSO_4_ solution [11]	*S. mutans*	Animal	Intervention: (5.0 mM, 1.0–5.0 mM) CuSO_4_ solution Control: water	Drinking water
CuSO_4_ solution [24]	*S. sobrinus*	Animal	Intervention: (300 ppm, 75–300 ppm) CuSO_4_ solution Control: water	Sugar
Cu_3_(PO_4_)_2_ solution [38]	*S. mutans*	In vitro	Intervention: Cu_3_(PO_4_)_2_ cement Control: conventional cement without Cu_3_(PO_4_)_2_	Restorative filler
CuF_2_ solution [36]	*S. mutans*	Animal	Intervention: 10 mM CuF_2_ solution Controls: CuSO_4_; NaF; water	Topical agent
CuCl_2_ solution [28]	*S. mutans*	In vitro	Intervention: 1% CuCl_2_ solution Control: water	Topical agent
CuI nanoparticles [15]	*S. mutans*	In vitro	Intervention: (1.0 mg/mL, 0.5–1.0 mg/mL) CuI-polyacrylic acid nanoparticles Control: conventional dental adhesive without CuI nanoparticles	Dental adhesive
CuI nanoparticles [30]	*S. mutans*	In vitro	Intervention: 0.263 wt% CuI-polyacrylic acid nanoparticles in glass ionomer Control: conventional glass ionomer without CuI nanoparticles	Restorative filler
CuI nanoparticles [44]	*S. mutans, L. acidophilus*	In vitro	Intervention: (5.0 μg/mL, 0.5–5.0 μg/mL) CuI nanoparticle dental adhesive Control: conventional dental adhesive without CuI nanoparticles	Dental adhesive

## References

[1] Petersen P.E. (2008). World Health Organization Global Policy for Improvement of Oral Health—World Health Assembly 2007. Int. Dent. J..

[2] Collaborators GDaIIaP (2018). Global, Regional, and National Incidence, Prevalence, and Years Lived with Disability for 354 Diseases and Injuries for 195 Countries and Territories, 1990–2017: A Systematic Analysis for the Global Burden of Disease Study 2017. Lancet.

[3] Selwitz R.H., Ismail A.I., Pitts N.B. (2007). Dental Caries. Lancet.

[4] Niu J.Y., Yin I.X., Wu W.K.K., Li Q.L., Mei M.L., Chu C.H. (2021). Antimicrobial Peptides for the Prevention and Treatment of Dental Caries: A Concise Review. Arch. Oral. Biol..

[5] Xue V.W., Yin I.X., Niu J.Y., Lo E.C.M., Chu C.H., Zhao I.S. (2022). Effects of a 445 Nm Diode Laser and Silver Diamine Fluoride in Preventing Enamel Demineralisation and Inhibiting Cariogenic Bacteria. J. Dent..

[6] Gadallah L.K., Safwat E.M., Saleh R.S., Azab S.M., Azab M.M. (2023). Effect of Silver Diamine Fluoride/Potassium Iodide Treatment on the Prevention of Dental Erosion in Primary Teeth: An in Vitro Study. BDJ Open.

[7] Yin I.X., Zhao I.S., Mei M.L., Lo E.C.M., Tang J., Li Q., So L.Y., Chu C.H. (2020). Synthesis and Characterization of Fluoridated Silver Nanoparticles and Their Potential as a Non-Staining Anti-Caries Agent. Int. J. Nanomed..

[8] Dollwet H.H.A., Sorenson J.R.J. (1985). Historic Uses of Copper Compounds in Medicine. Trace Elem. Med..

[9] O’Gorman J., Humphreys H. (2012). Application of Copper to Prevent and Control Infection. Where Are We Now?. J. Hosp. Infect..

[10] Meiers J.C., Schachtele C.F. (1984). The Effect of an Antibacterial Solution on the Microflora of Human Incipient Fissure Caries. J. Dent. Res..

[11] Afseth J., Amsbaugh S.M., Monell-Torrens E., Bowen W.H., Rölla G., Brunelle J., Li S., Dahl E. (1984). Effect of Topical Application of Copper in Combination with Fluoride in Drinking Water on Experimental Caries in Rats. Caries Res..

[12] Al-Hijazi A.Y., Hasan N., Nasr B.K., Jasim Al-Khafaji H.H., Al-Khafaji B., Abdah Alanssari B.F., Jalil A.T. (2023). Recent Advances in the Use of Inorganic Nanomaterials as Anti Caries Agents. Heliyon.

[13] Essa A.M., Khallaf M.K. (2016). Antimicrobial Potential of Consolidation Polymers Loaded with Biological Copper Nanoparticles. BMC Microbiol..

[14] Xu V.W., Nizami M.Z.I., Yin I.X., Lung C.Y.K., Yu O.Y., Chu C.H. (2022). Caries Management with Non-Metallic Nanomaterials: A Systematic Review. Int. J. Nanomed..

[15] Sabatini C., Mennito A.S., Wolf B.J., Pashley D.H., Renné W.G. (2015). Incorporation of Bactericidal Poly-Acrylic Acid Modified Copper Iodide Particles into Adhesive Resins. J. Dent..

[16] Liu R., Memarzadeh K., Chang B., Zhang Y., Ma Z., Allaker R.P., Ren L., Yang K. (2016). Antibacterial Effect of Copper-Bearing Titanium Alloy (Ti-Cu) against Streptococcus Mutans and Porphyromonas Gingivalis. Sci. Rep..

[17] Aguilar-Perez D., Vargas-Coronado R., Cervantes-Uc J.M., Rodriguez-Fuentes N., Aparicio C., Covarrubias C., Alvarez-Perez M., Garcia-Perez V., Martinez-Hernandez M., Cauich-Rodriguez J.V. (2020). Antibacterial Activity of a Glass Ionomer Cement Doped with Copper Nanoparticles. Dent. Mater. J..

[18] Pasha M., Muhammad N., Nayyer M., Bokhari J.H., Ashraf H., Safi S.Z., Kaleem M. (2020). Synthesis of an Anti-Cariogenic Experimental Dental Composite Containing Novel Drug-Decorated Copper Particles. Mater. Sci. Eng. C Mater. Biol. Appl..

[19] Imani M.M., Kiani M., Rezaei F., Souri R., Safaei M. (2021). Optimized Synthesis of Novel Hydroxyapatite/Cuo/Tio_2_ Nanocomposite with High Antibacterial Activity against Oral Pathogen Streptococcus Mutans. Ceram. Int..

[20] Mao M., Zhang W., Huang Z., Huang J., Wang J., Li W., Gu S. (2021). Graphene Oxide-Copper Nanocomposites Suppress Cariogenic Streptococcus Mutans Biofilm Formation. Int. J. Nanomed..

[21] Bang S.J., Jun S.K., Kim Y.J., Ahn J.Y., Vu H.T., Mandakhbayar N., Han M.R., Lee J.H., Kim J.B., Kim J.S. (2022). Characterization of Physical and Biological Properties of a Caries-Arresting Liquid Containing Copper Doped Bioglass Nanoparticles. Pharmaceutics.

[22] Li Q., Liu J., Xu Y., Liu H., Zhang J., Wang Y., Sun Y., Zhao M., Liao L., Wang X. (2022). Fast Cross-Linked Hydrogel as a Green Light-Activated Photocatalyst for Localized Biofilm Disruption and Brush-Free Tooth Whitening. ACS Appl. Mater. Interfaces.

[23] Afseth J., Amsbaugh S.M., Monell-Torrens E., Bowen W.H., Rølla G., Brunelle J., Dahl E. (1984). Effect of Copper Applied Topically or in Drinking Water on Experimental Caries in Rats. Caries Res..

[24] Rosalen P.L., Bowen W.H., Pearson S.K. (1996). Effect of Copper Co-Crystallized with Sugar on Caries Development in Desalivated Rats. Caries Res..

[25] Abdullah A.Z., Strafford S.M., Brookes S.J., Duggal M.S. (2006). The Effect of Copper on Demineralization of Dental Enamel. J. Dent. Res..

[26] Argueta-Figueroa L., Morales-Luckie R.A., Scougall-Vilchis R.J., Olea-Mejia O.F. (2014). Synthesis, Characterization and Antibacterial Activity of Copper, Nickel and Bimetallic Cu-Ni Nanoparticles for Potential Use in Dental Materials. Prog. Nat. Sci. -Mater. Int..

[27] Bakale R.P., Pathan A.H., Naik G.N., Machakanur S.S., Mangannavar C.V., Muchchandi I.S., Gudasi K.B. (2014). Synthesis and Characterization of Transition Metal Complexes of Hydrochloride Salt of 3-Chlorobenzaldehyde Hydralazine Hydrazone: A New Class of Possible Anti-Cariogenic Agents. Appl. Organomet. Chem..

[28] Girenes G., Ulusu T. (2014). An in Vitro Evaluation of the Efficacy of a Novel Iontophoresis Fluoride Tray on Remineralization. J. Clin. Exp. Dent..

[29] Ramazanzadeh B., Jahanbin A., Yaghoubi M., Shahtahmassbi N., Ghazvini K., Shakeri M., Shafaee H. (2015). Comparison of Antibacterial Effects of Zno and Cuo Nanoparticles Coated Brackets against Streptococcus Mutans. J. Dent..

[30] Renné W.G., Lindner A., Mennito A.S., Agee K.A., Pashley D.H., Willett D., Sentelle D., Defee M., Schmidt M., Sabatini C. (2017). Antibacterial Properties of Copper Iodide-Doped Glass Ionomer-Based Materials and Effect of Copper Iodide Nanoparticles on Collagen Degradation. Clin. Oral. Investig..

[31] Covarrubias C., Trepiana D., Corral C. (2018). Synthesis of Hybrid Copper-Chitosan Nanoparticles with Antibacterial Activity against Cariogenic Streptococcus Mutans. Dent. Mater. J..

[32] Javed R., Rais F., Kaleem M., Jamil B., Ahmad M.A., Yu T., Qureshi S.W., Ao Q. (2021). Chitosan Capping of Cuo Nanoparticles: Facile Chemical Preparation, Biological Analysis, and Applications in Dentistry. Int. J. Biol. Macromol..

[33] Altankhishig B., Matsuda Y., Nagano-Takebe F., Okuyama K., Yamamoto H., Sakurai M., Naito K., Hayashi M., Sano H., Sidhu S.K. (2022). Potential of Fluoride-Containing Zinc Oxide and Copper Oxide Nanocomposites on Dentin Bonding Ability. Nanomaterials.

[34] Ashour A.A., Felemban M.F., Felemban N.H., Enan E.T., Basha S., Hassan M.M., Gad El-Rab S.M.F. (2022). Comparison and Advanced Antimicrobial Strategies of Silver and Copper Nanodrug-Loaded Glass Ionomer Cement against Dental Caries Microbes. Antibiotics.

[35] Fan D.Y., Yi Z., Feng X., Tian W.Z., Xu D.K., Valentino A.M.C., Wang Q., Sun H.C. (2022). Antibacterial Property of a Gradient Cu-Bearing Titanium Alloy by Laser Additive Manufacturing. Rare Met..

[36] Maltz M., Emilson C.G. (1988). Effect of Copper Fluoride and Copper Sulfate on Dental Plaque, Streptococcus Mutans and Caries in Hamsters. Scand. J. Dent. Res..

[37] Brookes S.J., Shore R.C., Robinson C., Wood S.R., Kirkham J. (2003). Copper Ions Inhibit the Demineralisation of Human Enamel. Arch. Oral. Biol..

[38] Foley J., Blackwell A. (2003). Ion Release from Copper Phosphate Cement and Influence on Streptococcus Mutans Growth in Vitro: A Comparative Study. Caries Res..

[39] Thneibat A., Fontana M., Cochran M.A., Gonzalez-Cabezas C., Moore B.K., Matis B.A., Lund M.R. (2008). Anticariogenic and Antibacterial Properties of a Copper Varnish Using an in Vitro Microbial Caries Model. Oper. Dent..

[40] Amiri M., Etemadifar Z., Daneshkazemi A., Nateghi M. (2017). Antimicrobial Effect of Copper Oxide Nanoparticles on Some Oral Bacteria and Candida Species. J. Dent. Biomater..

[41] Glauser S., Astasov-Frauenhoffer M., Müller J.A., Fischer J., Waltimo T., Rohr N. (2017). Bacterial Colonization of Resin Composite Cements: Influence of Material Composition and Surface Roughness. Eur. J. Oral. Sci..

[42] Gutiérrez M.F., Bermudez J., Dávila-Sánchez A., Alegría-Acevedo L.F., Méndez-Bauer L., Hernández M., Astorga J., Reis A., Loguercio A.D., Farago P.V. (2019). Zinc Oxide and Copper Nanoparticles Addition in Universal Adhesive Systems Improve Interface Stability on Caries-Affected Dentin. J. Mech. Behav. Biomed. Mater..

[43] Lan Y., Yang J., Liu X., Zhao H., Zhang X., Yin X., Yang C., Yang K., Liu Y. (2022). Inhibition Efficiency of 304-Cu Stainless Steel against Oral Bacterial Biofilm. J. Appl. Biomater. Funct. Mater..

[44] Mennito A.S., Schmidt M., Lane A., Kelly A., Sabatini C., Renne W., Evans Z. (2022). Assessing the Antimicrobial Properties of Copper-Iodide Doped Adhesives in an in Vitro Caries Model: A Pilot Study. Contemp. Clin. Dent..

[45] Zhang Y.X., Liu W.Z., Huang Y.M., Wang Y.H., Chen X.Y., Chen Z. (2022). Bacterial Biofilm Microenvironment Responsive Copper-Doped Zinc Peroxide Nanocomposites for Enhancing Chemodynamic Therapy. Chem. Eng. J..

[46] Matsuda Y., Okuyama K., Yamamoto H., Fujita M., Abe S., Sato T., Yamada N., Koka M., Sano H., Hayashi M. (2019). Antibacterial Effect of a Fluoride-Containing Zno/Cuo Nanocomposite. Nucl. Instrum. Methods Phys. Res. Sect. B-Beam Interact. Mater. At..

[47] Ren G., Hu D., Cheng E.W., Vargas-Reus M.A., Reip P., Allaker R.P. (2009). Characterisation of Copper Oxide Nanoparticles for Antimicrobial Applications. Int. J. Antimicrob. Agents.

[48] Xu V.W., Nizami M.Z.I., Yin I.X., Yu O.Y., Lung C.Y.K., Chu C.H. (2022). Application of Copper Nanoparticles in Dentistry. Nanomaterials.

[49] Makkar H., Patri G. (2017). Fabrication and Appraisal of Poly (Lactic-Co-Glycolic Acid)—Moxifloxacin Nanoparticles Using Vitamin E-Tpgs: A Potential Intracanal Drug Delivery Agent. J. Clin. Diagn. Res..

[50] Yin I.X., Zhang J., Zhao I.S., Mei M.L., Li Q., Chu C.H. (2020). The Antibacterial Mechanism of Silver Nanoparticles and Its Application in Dentistry. Int. J. Nanomed..

[51] Liu H.L., Nosheen F., Wang X. (2015). Noble Metal Alloy Complex Nanostructures: Controllable Synthesis and Their Electrochemical Property. Chem. Soc. Rev..

[52] Rawashdeh R.Y., Qabaja G., Albiss B.A. (2023). Antibacterial Activity Of multi-Metallic (Ag–Cu–Li) Nanorods with Different Metallic Combination Ratios against Staphylococcus Aureus. BMC Res. Notes.

[53] Ognik K., Cholewińska E., Juśkiewicz J., Zduńczyk Z., Tutaj K., Szlązak R. (2019). The Effect of Copper Nanoparticles and Copper (Ii) Salt on Redox Reactions and Epigenetic Changes in a Rat Model. J. Anim. Physiol. Anim. Nutr..

[54] Street R.A., Kabera G.M., Connolly C. (2017). Copper Sulphate Use in South African Traditional Medicine. Environ. Geochem. Health.

